# Pro-metastatic and mesenchymal gene expression signatures characterize circulating tumor cells of neuroblastoma patients with bone marrow metastases and relapse

**DOI:** 10.3389/fonc.2022.939460

**Published:** 2022-09-13

**Authors:** Amos H. P. Loh, Clara Angelina, Meng Kang Wong, Sheng Hui Tan, Sarvesh A. Sukhatme, Trifanny Yeo, Su Bin Lim, York Tien Lee, Shui Yen Soh, Wing Leung, Kenneth T. E. Chang, Yong Wei Chua, Syed M. F. Alkaff, Tony K. H. Lim, Chwee Teck Lim, Zhi Xiong Chen

**Affiliations:** ^1^ VIVA-KKH Paediatric Brain and Solid Tumour Programme, Children’s Blood and Cancer Centre, KK Women’s and Children’s Hospital, Singapore, Singapore; ^2^ Department of Paediatric Surgery, KK Women’s and Children’s Hospital, Singapore, Singapore; ^3^ Duke NUS Medical School, Singapore, Singapore; ^4^ Department of Physiology, Yong Loo Lin School of Medicine, National University of Singapore, Singapore, Singapore; ^5^ Mechanobiology Institute of Singapore, National University of Singapore, Singapore, Singapore; ^6^ Department of Biomedical Engineering, National University of Singapore, Singapore, Singapore; ^7^ NUS Graduate School for Integrative Sciences and Engineering, National University of Singapore, Singapore, Singapore; ^8^ Department of Paediatric Subspecialties Haematology/Oncology Service, KK Women’s and Children’s Hospital, Singapore, Singapore; ^9^ Department of Pathology and Laboratory Medicine, KK Women’s and Children’s Hospital, Singapore, Singapore; ^10^ Department of Anatomic Pathology, Singapore General Hospital, Singapore, Singapore; ^11^ Institute of Health Innovation and Technology, National University of Singapore, Singapore, Singapore; ^12^ National University Cancer Institute, National University Health System, Singapore, Singapore; ^13^ NUS Centre for Cancer Research, Yong Loo Lin School of Medicine, National University of Singapore, Singapore, Singapore

**Keywords:** circulating tumor cells, neuroblastoma, minimal residual disease, microfluidic, bone marrow metastasis

## Abstract

Existing marker-based methods of minimal residual disease (MRD) determination in neuroblastoma do not effectively enrich for the circulating disease cell population. Given the relative size differential of neuroblastoma tumor cells over normal hematogenous cells, we hypothesized that cell size-based separation could enrich circulating tumor cells (CTCs) from blood samples and disseminated tumor cells (DTCs) from bone marrow aspirates (BMA) of neuroblastoma patients, and that their gene expression profiles could vary dynamically with various disease states over the course of treatment. Using a spiral microfluidic chip, peripheral blood of 17 neuroblastoma patients at 3 serial treatment timepoints (diagnosis, n=17; post-chemotherapy, n=11; and relapse, n=3), and bone marrow samples at diagnosis were enriched for large intact circulating cells. Profiling the resulting enriched samples with immunohistochemistry and mRNA expression of 1490 cancer-related genes *via* NanoString, 13 of 17 samples contained CTCs displaying cytologic atypia, TH and PHOX2B expression and/or upregulation of cancer-associated genes. Gene signatures reflecting pro-metastatic processes and the neuroblastoma mesenchymal super-enhancer state were consistently upregulated in 7 of 13 samples, 6 of which also had metastatic high-risk disease. Expression of 8 genes associated with PI3K and GCPR signaling were significantly upregulated in CTCs of patients with bone marrow metastases versus patients without. Correspondingly, in patients with marrow metastases, differentially-expressed gene signatures reflected upregulation of immune regulation in bone marrow DTCs versus paired CTCs samples. In patients who later developed disease relapse, 5 genes involved in immune cell regulation, JAK/STAT signaling and the neuroblastoma mesenchymal super-enhancer state (OLFML2B, STAT1, ARHGDIB, STAB1, TLR2) were upregulated in serial CTC samples over their disease course, despite urinary catecholamines and bone marrow aspirates not indicating the disease recurrences. In summary, using a label-free cell size-based separation method, we enriched and characterized intact circulating cells in peripheral blood indicative of neuroblastoma CTCs, as well as their DTC counterparts in the bone marrow. Expression profiles of pro-metastatic genes in CTCs correlated with the presence of bone marrow metastases at diagnosis, while longitudinal profiling identified persistently elevated expression of genes in CTCs that may serve as novel predictive markers of hematogenous MRD in neuroblastoma patients that subsequently relapse.

## Introduction

Neuroblastoma is the commonest extracranial malignancy of childhood and responsible for a disproportionate number of deaths from childhood cancer. Nearly 60% of neuroblastomas relapse in distant sites (1), most commonly bone marrow. Disease relapse is thought to arise from undetected, chemo-resistant cells. Yet, current treatment-response evaluations in neuroblastoma do not consider MRD for treatment allocation, particularly of bone marrow and blood, unlike in many hematological and adult cancers where this is a routine part of clinical treatment protocols (2–5). As the thoroughfare for cellular trafficking, these compartments are thought to harbor micrometastases that seed distant sites. Their prognostic significance has been demonstrated in various adult malignancies and pediatric leukemia (6). However, existing PCR-based approaches to determine MRD in neuroblastoma provide limited actionable biological information and are unable to enrich for the cells in question (7–11).

More recently, single cell capture techniques have allowed CTCs to be enriched from peripheral blood. However, as most CTC capture platforms employ affinity-binding methods, they are limited by low throughput, cell viability and an inherent selection bias (12–15). Thus, non-affinity-binding methods may facilitate enrichment for a population of intact, viable CTCs in a high-throughput manner. Size-based separation methods have also identified circulating cells undergoing epithelial to mesenchymal transition, with biological characteristics of malignancy but lacking known surface epithelial markers like EpCam (15). Using capture-based methods, CTCs expressing neurogenic markers have been isolated from blood of neuroblastoma patients and shown to correlate with relapse and bone marrow metastases (16–18). Yet, as neuroblastoma tumor cells are mostly larger than normal blood cells (~20μm vs ~12μm), and have been found in peripheral blood samples (19), this suggests that they may be selectively concentrated by size-based separation (20). A spiral microchannel biochip utilizing inertial microfluidics and inherent centrifugal forces for size-based separation of CTCs from blood has been successfully employed in various cancers (21–25). We hypothesized that this high-throughput label-free method could enrich CTCs from blood samples and DTCs from bone marrow aspirates (BMA) of neuroblastoma patients, and that gene expression of these cells would vary dynamically with various disease states over the course of treatment.

In this study, we enriched CTCs and DTCs that expressed neuroblastoma markers on immunohistochemistry and quantitative reverse transcription PCR (RT-qPCR). We identified distinct CTC and DTC expression signatures that distinguish neuroblastoma patients with bone marrow metastases at initial diagnosis, and that persist in patients with subsequent relapse.

## Materials and methods

### Patients and samples

**Figure 1:**
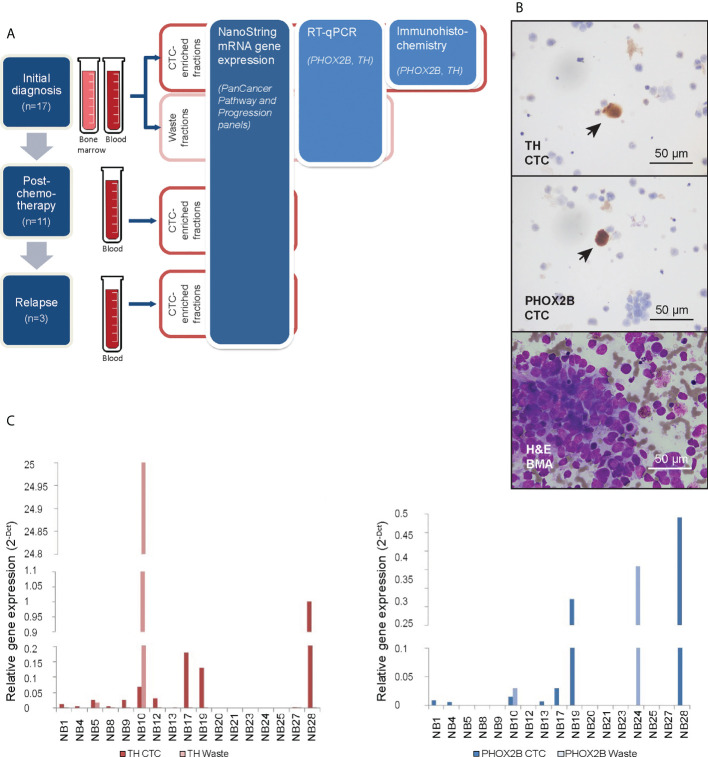
Neuroblastoma CTCs expressing characteristic markers are isolated using cell size-based separation. **(A)** Schema of experimental design and 3 serial timepoints where blood and/or bone marrow samples were obtained from patients for CTC enrichment. **(B)** Representative photomicrographs of cytospots of CTC-enriched fractions demonstrating PHOX2B- and TH-positivity of isolated large, atypical cells on immunohistochemical staining (arrows; NBL20 at initial diagnosis and NBL10 following induction chemotherapy, respectively; scale bar: 50 µm), and corresponding bone marrow aspirate with neuroblastoma tumor cell infiltration for size comparison (NBL20 at initial diagnosis; H&E, scale bar: 50 µm). **(C)** Relative gene expression of PHOX2B and TH in CTC-enriched and waste fractions from blood samples taken at diagnosis.

Following informed consent under an institutional review board-approved protocol (SHS/2016/2022), neuroblastoma patients treated at KK Women’s and Children’s Hospital were recruited at initial diagnosis. Tumor, venous blood and BMA samples were obtained at diagnosis, after induction chemotherapy following ANBL0032 protocol, and at relapse (**Figure 1A**). Demographic, disease, treatment and outcome data were obtained from the Singapore Childhood Cancer Registry (SCCR). All tumor and BMA smears were centrally reviewed by a senior pediatric pathologist and evaluated according to International Neuroblastoma Response Criteria standards (26).

At respective timepoints, at least 6ml of venous blood and 3ml of BMA were drawn and collected using K2-EDTA vacutainer^®^ tubes (BD, Singapore) or Cell-Free DNA BCT tubes (Streck, USA) and processed on the same working day using the ClearCell^®^ FX system (Biolidics, Singapore), as described (27, 28). Separate CTC-enriched and CTC-depleted (waste) fractions were obtained. CTC-enriched fractions were separated equally for downstream gene expression analysis and immunocytochemistry to verify their expression of established neuroblastoma markers. Cytospots were created from half of each CTC-enriched fraction for immunohistochemistry, and mRNA was purified from the other half for gene expression analysis. For gene expression analysis of CTCs and cell lines, RNA was extracted using the RNeasy micro kit (Qiagen, Germany). RNA was quantified using nanodrop and stored at -80°C. For immunohistochemistry, the apportioned CTC output was fixed in Shandon Cytospin Collection Fluid (Fisher Scientific, Inc.) and cytospots stained for PHOX2B, TH and GD2 synthase.

For initial evaluation of cell separation efficacy and subsequent gene expression analysis, 1mL whole blood samples from 3 anonymized age-matched healthy controls were obtained from the Department of Pathology and Laboratory Medicine, KK Women’s and Children’s Hospital, under the same research protocol.

### Cell lines

Human neuroblastoma cell lines NB1, CHP212, SK-N-SH, NLF (RRID: CVCL_1440, CVCL_1125, CVCL_D044, CVCL_E217) and human gastric cancer cell line AGS (RRID: CVCL_0139) were maintained in RPMI-1640 (Hyclone) containing 10% fetal bovine serum (FBS; Hyclone). Kelly was maintained in RPMI-1640 (Hyclone) with HEPES containing 10% FBS. IMR32 was maintained in MEM/EBSS (Hyclone) containing 1% NEAA, 1% sodium pyruvate and 10% FBS. BE2C was maintained in DMEM/F12 (Hyclone) containing 10% FBS. All cells were obtained from America Type Culture Collection (ATCC) and cultured in a 37°C, 5% CO_2_ humidified incubator.

### Single-cell isolation using a microfluidic device

Normal blood samples spiked with NLF cells were subjected to 1% paraformaldehyde (PFA) fixation and staining with Anti-Human CD45-PE (Miltenyi Biotec, Germany, RRID : AB_2725946) and Hoechst 33342 (Trihydrochloride, Trihydrate, Life Technologies, CA, USA, RRID : AB_10626776) prior to loading into the microfluidic device. Having 10 single-cell capture chambers, the device was mounted on a microscope (Olympus BX61, Japan, RRID : SCR_020343) for isolation of single CTCs based on immunofluorescence and morphology. Two syringe pumps (Chemyx Fusion 200, TX, USA) were used to maintain constant flow rates (i.e., cell flow to sheath flow = 10 μl/min: 30μl/min). Hoechst^+^/CD45^-^ cell (i.e., CTC) and Hoechst^+^/CD45^+^ cell (i.e., WBC) in the capture chamber were ejected into the *recovery* and *recycling* port, respectively.

### Single-cell lysis and cDNA generation

Each single CTC was transferred to 0.2 ml PCR tube and subjected to lysis and RNA extraction according to the manufacturer’s specifications (Single Cell Lysis Kit, Thermo Fisher Scientific, MA, USA). 2.5 μM oligo (dT) primers and 0.5 mM dNTP Mix (all Life Technologies, Singapore) were added into the lysed CTC sample, which was subsequently incubated at 65°C for 5 min and cooled on ice for at least 1 min. 1x first-strand buffer, 5 mM DTT, 10 U RNaseOUT Recombinant RNase Inhibitor, and 50 U SuperScript III RT (all Life Technologies, Singapore) were used, made up to a final volume of 20 μl in nuclease-free water. The final product was incubated at 25°C for 5 min, 55°C for 60 min, and 85°C for 5 min for reverse transcription on a C1000TM Thermal Cycler (Bio-Rad, Hercules, USA).

### Target-specific preamplification

Prior to preamplification, 1 μM primer mix comprising PHOX2B, TH, GD2 synthase, β2 microglobulin, GAPDH and UBB gene primers were prepared by adding 1 μl of 100 μM forward gene primer and 1 μl of 100 μM reverse gene primer up to a final volume of 100 μl in nuclease-free water. 1x PCRBIO Ultra Mix (PCR Biosystems Ltd, London, UK), 100 nM of each primer, and 10 μl of the reverse-transcribed products were added to a final volume of 20 μl in nuclease-free water. The final product was incubated at 95°C for 10 min, followed by 25 cycles of 95°C for 20 sec, 60°C for 1 min and 72°C for 20 sec with an addition of 1 cycle of 72°C for 7 min on a C1000™ Thermal Cycler (Bio-Rad, Hercules, USA). The amplified products were purified prior to quantitation using Agencourt AMPure XP beads (Beckman Coulter, IN, USA) according to the manufacturer’s recommendations.

### Real-time quantitative PCR

1x FastStart SYBR Green Master mix (Roche), 300 nM of forward and reverse gene primer (Integrated DNA Technologies), and 1 μl of eluted DNA product were added to a final volume of 10 μl in nuclease-free water. The final product was incubated at 95°C for 10 min, followed by 40 cycles of 95°C for 20 sec, 55°C for 30 sec and 72°C for 20 sec with an addition of 1 cycle of 72°C for 7 min on a CFX96 Real-Time PCR Detection System (Bio-Rad, Hercules, USA). Two housekeeping genes (i.e., GADPH and UBB) were used for normalization of expression data. Each experiment was performed in duplicate.

GAPDH forward: CAAGCTCATTTCCTGGTATGAC
GAPDH reverse: CAGTGAGGGTCTCTCTCTTCCT
UBB forward: GCTTTGTTGGGTGAGCTTGT
UBB reverse: CGAAGATCTGCATTTTGACCT

### Gene expression analysis using real-time quantitative PCR

cDNA was synthesized from 10 ng of total RNA of each sample (Promega, USA). Two µl of cDNA in triplicate was used for real-time quantitative PCR (RT-qPCR) in 384-well plate (Bio-Rad, USA) and performed on LightCycler^®^ 480 System (Roche, Switzerland, RRID : SCR_020502). PHOX2B (forward primer; 5’-GGCTTCCAGTATAACCCGATAAG-3’, reverse primer; 5’-TGGTCCGTGAAGAGTTTGTAAG-3’), tyrosine hydroxylase (TH) (forward primer; 5’-ATTGCTGAGATCGCCTTCCA -3’, reverse primer; 5’-AATCTCCTCGGCGGTGTACTC -3’), GD2 synthase (forward primer; 5’-GACAAGCCAGAGCGCGTTA-3’, reverse primer; 5’-TACTTGAGACACGGCCAGGTT-3’), and β2 microglobulin (forward primer; 5’-GAGTATGCCTGCCGTGTG-3’, reverse primer; 5’-AATCCAAATGCGGCATCT-3’), primers were designed as previously described and purchased from Sigma Aldrich (Merck, Germany) (29–31). Primers were checked for specificity using Primer-BLAST (32). The samples were considered positive if at least two of the three quantification cycle (Ct) values were lower than 40. Positive results of RT-qPCR analysis were expressed as ΔΔCt values using β2-microglobulin as endogenous reference mRNA, and the NB1 and AGS cell lines as the exogenous reference samples.

### Immunohistochemistry

CTC and DTC cytospots were fixed in 10% buffered formalin (Leica Biosystems, Richmond VA), and stained with PHOX2B (1:100) (ab183741, Abcam, RRID : AB_2857845) and TH (1:3200) (66334-1-Ig, Proteintech, RRID : AB_2881714) with the BOND-III Automated IHC stainer (Leica Biosystems, USA), using manufacturers’ default automated staining protocol, as follows: pre-treatment unmasking with BOND epitope retrieval solution 2 (for PHOX2B) or 1 (for TH) (Cat. AR9640, AR9961, Leica Biosystems, USA), wash with absolute alcohol and Bond Wash Solution, staining with primary antibodies and detection using Bond™ Polymer Refine Detection (Cat. DS9800, Leica Biosystems, USA). After staining, slides were dehydrated in absolute alcohol, cleared with xylene and mounted in DEPEX medium.

### NanoString gene expression analysis of clinical samples

Samples (1ng) were amplified using nCounter Low RNA Input Amplification Kit followed by multiplexed target enrichment according to manufacturer’s instruction and underwent 17-hour hybridization and post-hybridization high-sensitivity cleanup with the nanoString nCounter Prep Station (nanoString Technologies, USA, RRID : SCR_021712), and automated counting using the nCounter Digital Analyzer. Additional probes were added to the PanCancer Pathway panel encoding for KIF1Bβ, PHOX2B, TH, GD2 synthase, CHD5, LIN28B, CASZ1, BARD1, LMO1, and TP73; and to the Progression panel for KIF1Bβ, PHOX2B, TH, GD2 synthase, CHD5, CHRNA3, PTPN14, GAP43, DCX and DDC. Samples were hybridized for 17 hours and underwent nanoString nCounter gene expression assay, according to manufacturer’s instructions (nanoString Technologies, USA, RRID : SCR_021712). Genes with fewer than the recommended minimum background threshold of 20 probe counts were filtered out. Custom gene signatures comprising genes of the ADR and MES neuroblastoma super enhancer state found within the PanCancer panels were defined and used to calculate custom ADR and MES signature scores across both panels. Raw data was analyzed using nSolver™ software (nanoString Technologies, USA, RRID : SCR_003420) under standard settings and normalized against manufacturer’s respective default housekeeping genes.

Pathway scores were generated using default annotations of the NanoString nSolver Advanced Analysis v2.0.115. Pathway scores are calculated as the first principal component of the pathway genes’ normalized expression, with a higher score indicating increased expression in the majority of pathway genes.

### Statistical analysis

Continuous variables were compared between clinical subgroups using one-way ANOVA. Univariate statistical significance was defined as a P-value of <0.05.

Differential expression analyses were performed using NanoString nSolver Advanced Analysis v2.0.115 and DESeq2 (RRID : SCR_000154) v.1.28.1 on R v.4.0.1 (33), and pathway enrichment analysis with default NanoString PanCancer signatures and the custom neuroblastoma gene set as previously described (34).

Statistical over-representation testing was performed using Protein ANalysis THrough Evolutionary Relationships annotations (PANTHER (RRID : SCR_004869), v.14) (35). Fisher exact test was used to compare the 46-gene bone marrow metastasis CTC signature and corresponding log_2_ fold-change values against a reference human genome. FDR was calculated using Benjamini-Hochberg procedure and an adjusted p-value of 0.05 was considered significant.

Since the counts of default housekeeping genes varied substantially between timepoints, in order to compare gene expression over multiple timepoints, the most stably-expressed housekeeping genes (*NOL7*, *COG7*, *NUBP1*, *DDX50* and *USP39)* were selected based on their respective average gene stability measure ~M (**Supplementary Data 1**), which was computed using geNorm geometric averaging as previously described (36). Analysis was performed on R v.4.0.1.

## Results

### Cells with cytologic atypia enriched from peripheral blood *via* cell size-based separation express characteristic neuroblastoma markers

To first evaluate the utility of size-based separation for neuroblastoma tumor cells, we used the ClearCell^®^ FX microfluidic device to recover neuroblastoma cells from normal blood samples spiked with the NLF neuroblastoma cell line. Large cells measuring 14-16 µm were captured (**Supplementary Figure 1A**), which expressed known neuroblastoma markers GD2 synthase, PHOX2B and TH (**Supplementary Figure 1B**) – the primers having been first verified by demonstrating their expression in 7 neuroblastoma cell lines. Since PHOX2B and TH showed the highest relative expression across the panel of cell lines compared to non-neuroblastoma cell line AGS (**Supplementary Figure 1C**), the separation method and primers were then used to discriminate neuroblastoma from blood cells in clinical samples.

We obtained peripheral blood samples from 17 consecutive neuroblastoma patients at initial diagnosis, as well as bone marrow aspirates from 4 of these patients (**Supplementary Table 1**). In 11 patients, blood samples were also drawn after induction chemotherapy and in 3 patients also during subsequent disease relapse. Each sample was enriched for CTCs using a spiral microchannel biochip and characterized accordingly (**Figure 1A**).

From half of each CTC-enriched fraction, cytospots were generated. Immunohistochemical staining showed large cells with cytological atypia that expressed PHOX2B and TH (**Figure 1B**). Mean diameter of CTCs on cytospots of samples obtained at initial diagnosis was 16.2 ± 8.0µm (n=78 cells). To explore the clinical relevance of their relative abundance in the clinical samples, we counted the number of cells with cytologic atypia in the cytospots of the 17 patients and correlated the cell counts with clinical and pathological variables. There were significantly more cells with cytologic atypia in CTC-enriched fractions of patients with bone marrow metastases than those without (P=0.04) (**Supplementary Table 2**). This suggested the association of the presence of peripheral blood cells with cytologic atypia with a metastatic disease phenotype.

Next, the other half of the same CTC-enriched fractions were compared with corresponding waste eluent from the ClearCell FX enrichment process for expression of neuroblastoma markers. RT-qPCR analysis showed higher PHOX2B and TH expression in CTC-enriched fractions compared to corresponding waste fractions in n=6 and n=10 cases respectively (**Figure 1C**). This suggested that increased mRNA expression of PHOX2B and TH in CTC-enriched fractions was unlikely due to acellular sources such as cell-free DNA. However, since PHOX2B and TH were markers of the adrenergic (ADR) super-enhancer state, and were not universally expressed by neuroblastoma cells, a further understanding of the gene expression landscape of these neuroblastoma CTCs, including markers of the mesenchymal (MES) state, was required.

### Neuroblastoma CTCs predominantly display pro-metastatic and mesenchymal gene expression signatures

We next sought to identify other cancer-related genes expressed in neuroblastoma CTCs. From the CTC-enriched fractions of the 17 neuroblastoma patients and peripheral blood from 3 healthy controls, we profiled the expression of 1490 genes from the NanoString PanCancer panels and their associated PanCancer gene signature scores (**Supplementary Data 2**). Unsupervised clustering of mRNA gene expression profiles identified 2 groups with gene expression profiles either similar to or distinct from controls (**Supplementary Figures 2A, B**). In 4 samples (cases NB 8, 23, 24 and 28) where the gene expression profiles clustered closest with controls, atypical cells expressing PHOX2B or TH were also not detected on immunohistochemistry (**Supplementary Figure 2C**). This suggested that these samples with expression profiles similar to blood and without cells with cytologic atypia likely contained few or no CTCs. Conversely, the remaining samples contained non-hematogenous cells with cytological atypia and increased expression of multiple cancer-associated genes.

**Figure 2:**
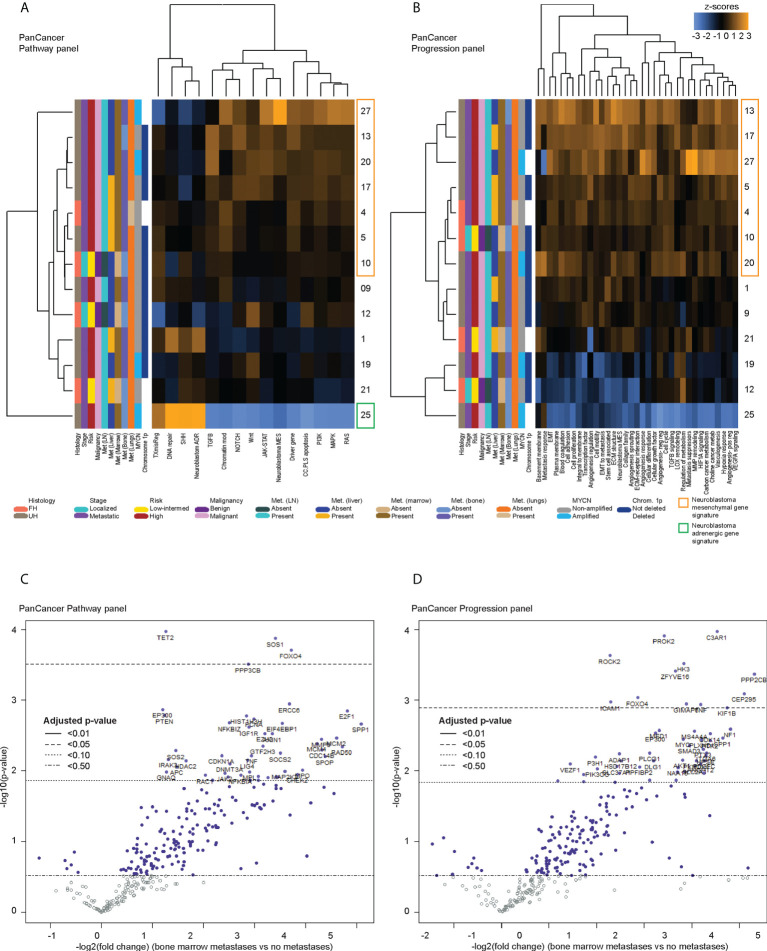
Differentially expressed genes in CTCs of neuroblastoma patients with bone marrow metastases. Heatmaps of unsupervised non-hierarchical clustering of the pathway signature scores of gene sets from **(A)** the PanCancer Pathway Panel, representing 13 cancer-associated canonical pathways, and **(B)** the PanCancer Progression Panel representing pathways involved in the cancer progression process, in CTC fractions of 13 neuroblastoma patients at initial diagnosis. Volcano plots of genes from the **(C)** PanCancer Pathway Panel, and **(D)** PanCancer Progression Panel that were significantly more upregulated in patients with bone marrow metastases than those without metastases, with multiple measures correction using the Benjamini-Hochberg method. Dot colors and reference lines indicate corresponding adjusted p-value thresholds.

Next, to determine differential gene expression signatures of CTC samples, PanCancer gene signature scores were calculated for the remaining 13 patients with samples that had a non-blood signature or where cells with cytologic atypia were seen on immunohistochemistry. Across both panels, multiple cancer-associated signaling pathways related to metastatic processes and a signature for the neuroblastoma mesenchymal state were consistently upregulated in 7 patients, had mixed expression in 5 patients, and were downregulated in 1 patient (**Figures 2A, B**). On the PanCancer Pathway panel, the latter patient had upregulation of the neuroblastoma adrenergic signature instead (**Figure 2A**); the adrenergic signature score could not be calculated in the Progression panel as probe counts were below the minimum threshold. Together, these results indicated that in a significant proportion of neuroblastoma patients, CTCs showed upregulation of a mesenchymal gene signature.

### Pro-metastatic genes are most significantly upregulated in CTCs of neuroblastoma patients with high risk metastatic disease

Since the presence of detectable CTCs was associated with a metastatic/mesenchymal disease phenotype, we then sought to understand the differential expression of unique genes in CTCs of patients with metastatic disease and other clinical risk features. First, using an intention-to-treat analysis approach, unsupervised clustering was performed to correlate gene expression against clinical variables for all 17 patients. Patients with relapse, and most patients with metastases showed upregulation of multiple genes from the NanoString PanCancer gene sets (**Supplementary Figure 3**). Among the 13 samples ascertained to contain CTCs, the earlier unsupervised clustering analysis showed that 6 of the 7 patients with consistent upregulation of PanCancer gene signatures and the neuroblastoma mesenchymal signature had metastatic high-risk disease, and metastases to lymph nodes and bone marrow (NB 4, 5, 13, 17, 20, 27) (**Figures 2A, B**). Together, these indicated that specific genes related to metastatic processes and the neuroblastoma mesenchymal state could be significantly differentially expressed in CTCs of patients with metastatic disease.

Since the bone marrow is the commonest site of distant metastasis and disease relapse in neuroblastoma, we focused on profiling the most dysregulated genes in the CTCs of patients with and without bone marrow metastases. Differential expression analysis was performed with FDR correction, comparing the 10 patients with bone marrow metastases against the other 3 without. This revealed 4 genes from the PanCancer Pathway panel (1.26–3.71 log_2_ fold change) and 12 genes from the PanCancer Progression panel (2.28–5.31 log_2_ fold change) that were significantly upregulated in the CTCs of patients with bone marrow metastases compared to those without (adjusted p-value <0.05) (**Figures 2C, D**) (**Supplementary Data 3**). In view of potential inter-panel differences in variance, we independently evaluated gene expression values of both NanoString PanCancer panels using DESeq2 with variance-stabilizing transformation (33). Applying similar FDR and adjusted p-value thresholds, in all, 2 of the above 4 PanCancer Pathway panel genes (SOS1 and FOXO4) and 6 of the above 12 PanCancer Progression panel genes (PROK2, C3AR1, ROCK2, ZFYVE16, HK3 and PPP2CB) were significantly differentially upregulated in patients with bone marrow metastases versus patients without bone marrow metastases (adjusted p-value <0.05) (**Table 1**; **Supplementary Figure 4**; **Supplementary Data 4**). Comparing the 8 genes against reference human genome using over-representation testing, they were enriched for REACTOME pathways related to signaling by FGFR1, FGFR3 and FGFR4 (log_2_ fold change >6.6) and GPCR downstream signaling (log_2_ fold change 4.4), and PANTHER pathways related to PI3K signaling (log_2_ fold change 6.5) (FDR adjusted P-value <0.05, Fisher exact test). Given their known function in tumor metastases (37, 38), the overexpression of these genes in the CTCs of neuroblastoma patients with bone marrow metastases indicated potential pro-metastatic processes in the CTCs of neuroblastoma patients that could play a role in development of bone marrow metastases.

**Table 1:** Genes significantly differentially expressed in patients with bone marrow metastases versus patients without bone marrow metastases, commonly identified by FDR and DESeq2 differential expression analyses.

Gene	FDR		DESeq2	
	Log_2_ fold change	Adj. P-value	Log_2_ fold change	Adj. P-value
PanCancer Pathway Panel				
SOS1	3.41	0.0213	2.80	0.029
FOXO4	3.71	0.0213	2.71	0.029
PanCancer Progression Panel				
PROK2	3.42	0.0202	3.53	0.001
C3AR1	4.53	0.0202	3.08	0.025
ROCK2	2.28	0.0238	2.20	0.032
ZFYVE16	3.66	0.0238	2.65	0.032
HK3	3.83	0.0238	2.50	0.041
PPP2CB	5.31	0.0238	2.52	0.041

Ordered by FDR P-value.

### DTCs in neuroblastoma bone marrow metastases express genes regulating immune response and stemness characteristics

**Figure 3:**
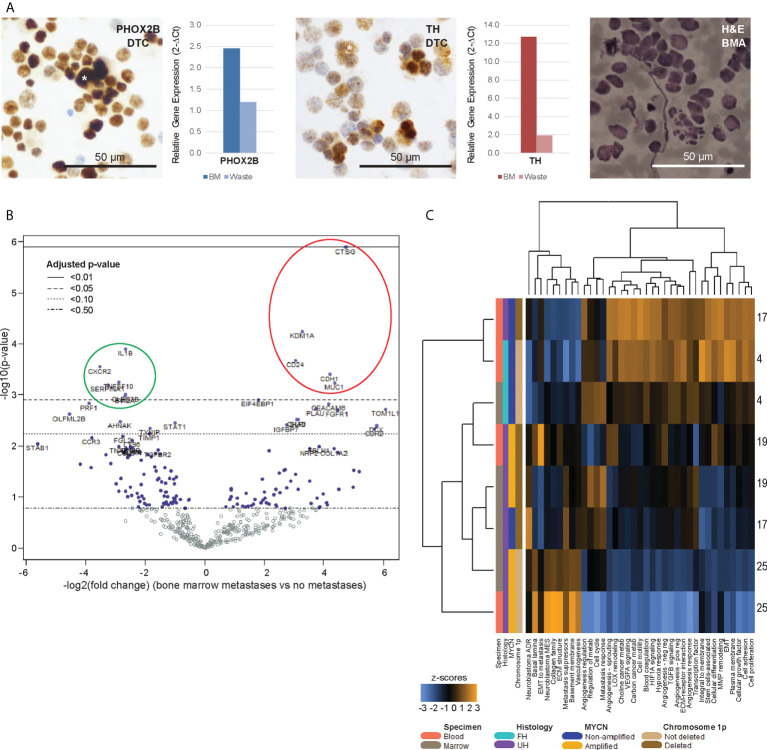
Bone marrow DTCs are isolated by size-based separation and express unique differentially expressed genes. **(A)** Representative photomicrographs of cytospots of DTC-enriched fractions from bone marrow aspirates of a patient with known bone marrow metastasis (NB9), showing nuclear immunoreactivity for PHOX2B and cytoplasmic staining for TH (asterisks). Corresponding relative gene expression of PHOX2B and TH in DTC-enriched bone marrow (BM) and waste fractions are shown alongside, as well as photomicrograph of BM aspirate showing infiltrating neuroblastoma tumor cells for size comparison (NB9, H&E, scale bar: 50 µm). **(B)** Volcano plot of genes from the PanCancer Progression Panel that were significantly upregulated (red) and downregulated (green) in bone marrow DTCs, compared to peripheral blood CTCs, in patients with known bone marrow metastases. Adjusted p-values are derived using Benjamini-Hochberg correction. Dot colors and reference lines indicate corresponding adjusted p-value thresholds. **(C)** Heatmap of signature scores of genes from the PanCancer Progression Panel in paired CTC and DTC samples from 4 patients with known bone marrow metastases, on unsupervised non-hierarchical clustering.

Next, we investigated the genes that were also significantly dysregulated in the DTCs of corresponding BMAs. Paired BMA samples drawn at initial diagnosis were similarly subjected to size-based enrichment with the ClearCell^®^ FX device, and immunohistochemical and gene expression of DTCs and CTCs from the same patients were compared. In 4 patients with clinically-proven metastatic disease in their bone marrow aspirates (NB4, NB17, NB19, NB25), DTCs stained positive for neuroblastoma immunohistochemical markers PHOX2B and TH, and DTC-enriched fractions from BMAs also expressed elevated levels of PHOX2B and TH on RT-qPCR (**Figure 3A**). Mean diameter of DTCs on cytospots of samples obtained at initial diagnosis was 21.5 ± 5.4µm (n=416 cells).

Comparing the differential gene expression profiles of the 4 CTC-DTC sample pairs from patients with bone marrow metastases, 5 genes (CD24, CDH1, CTSG, KDM1A, MUC1) were significantly upregulated in CTCs compared to DTCs, and 6 genes (CLEC2B, CXCR2 (IL8RB), EVI2A, IL1B, SERPINA1, TNFSF10) were significantly downregulated (adjusted p-value <0.05) (**Figure 3B**; **Supplementary Data 2**). Correspondingly, gene signatures for basal lamina, EMT to metastasis, collagen family, ECM structure, metastasis suppressors, basement membrane, vasculogenesis and the neuroblastoma mesenchymal super-enhancer state were upregulated in 3 of 4 DTC samples (**Figure 3C**). Together, these results indicated that DTCs and CTCs both expressed recognized neuroblastoma markers, and reflected an upregulation of innate immunity-associated cytokine signaling in DTCs, which could represent CTCs that have circulated into the bone marrow metastatic niche.

### Expression of genes related to the neuroblastoma mesenchymal super-enhancer state remain persistently elevated in CTCs of patients who relapse

Since bone marrow metastasis is closely related to clinical treatment failure in neuroblastoma, we studied the CTC expression profiles in 3 of 17 patients who subsequently relapsed. At initial diagnosis, CTC gene expression of the 2 patients with the shortest times to relapse (cases NB1 and 25) clustered separately from the third relapse patient who had a more protracted disease course (case NB13) and the non-relapse patients, with significant upregulation in a unique set of genes (**Supplementary Figures 2A, B**, **3**). Thus, we sought to understand if disease relapse might be associated with dysregulation of CTC genes at initial diagnosis or over the disease course.

**Figure 4:**
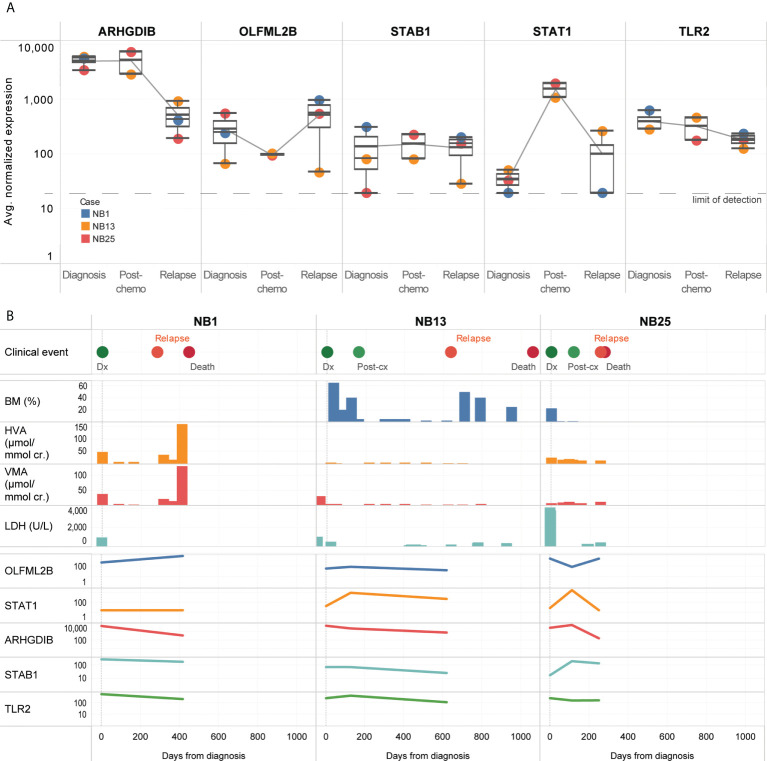
CTCs of relapse patients show persistent upregulation of genes related to interleukin and JAK/STAT signaling. **(A)** Boxplots showing distribution of normalized gene expression of 5 genes which were persistently elevated in 3 patients with disease relapse, at diagnosis, post-chemotherapy and relapse. **(B)** Graphical time course illustration of disease markers in 3 patients with disease relapse. Top panel: clinical events. Middle panel: serial values of standard clinical markers of disease including percentage of tumor involvement of bone marrow trephines, urinary catecholamines and serum tumor markers (BM: bone marrow, HVA: homovanillic acid, VMA: vanillylmandelic acid, LDH: lactate dehydrogenase). Lower panel: relative expression of 5 genes persistently elevated in CTCs of relapse patients.

Five genes (OLFML2B, STAT1, ARHGDIB, STAB1, TLR2) remained persistently upregulated despite treatment (**Figure 4A**). Among the candidate genes identified to be upregulated at diagnosis or over time, OLFML2B and STAT1 were known to be markers of the mesenchymal super-enhancer state. These remained elevated even though standard disease markers of bone marrow aspirate cytopathology, urinary catecholamines, and serum LDH did not consistently show a rise before the diagnosis of disease relapse (**Figure 4B**). Patient NB1 abandoned therapy and returned with liver metastases and elevated urinary catecholamines – at relapse, OLFML2B and STAT1 expression levels rose. Patients NB13 and NB25 relapsed on treatment after initial remission – in both, multiple genes showed increased expression, while routine urinary markers did not rise (**Figure 4B**). These anecdotal cases demonstrated that in CTCs of neuroblastoma patients with disease relapse, selected genes related to the mesenchymal super-enhancer state were upregulated at diagnosis, and remained so throughout treatment course, highlighting their potential utility to be studied as markers of persistent disease activity.

## Discussion

Using label-free size-based cell separation, we demonstrated for the first time the enrichment of intact cells from peripheral blood of neuroblastoma patients that displayed cytologic and gene expression characteristics of CTCs. We identified significantly dysregulated cancer-associated genes characterizing these CTCs and corresponding DTCs of patients with bone marrow metastasis at diagnosis, and showed that the gene expression profile of neuroblastoma CTCs is modulated temporally in response to systemic therapy. In a pilot set of patients who developed disease relapse, 5 genes remained persistently elevated in their CTC samples, suggesting possible subclinical persistence of disease that was not otherwise detected by conventional disease markers.

Measures of neuroblastoma MRD have been proposed in cell-free DNA or mRNA of peripheral blood (39, 40), and BMA (41, 42), and associated with unfavorable prognosis if detected at completion of therapy (39), or in contaminated peripheral blood stem cell harvests (43, 44). Attempts to enrich neuroblastoma CTCs have been largely limited to capture-based methods relying on cellular expression of TH, PHOX2B, NCAM and GD2 synthase (8, 16, 18), though these inherently introduce a selection bias. Proposed flow cytometric methods using CD45-negative gating suffer from significant false positivity (45, 46), while immunocytology is ineffective for hypocellular samples or those with clusters, as is often seen in peripheral blood or BMA (47). These limit the clinical usefulness of these MRD measures as actionable prognostic biomarkers (44, 47), and were also demonstrated in our findings. Instead, dysregulated mRNA expression of selected prognostic genes of neuroblastoma CTCs may represent more biologically-relevant markers of MRD (3, 48) in the intact CTCs captured *via* our unbiased label-free system.

In CTCs of a set of relapsed patients, we identified a set of persistently-elevated genes with known associations with metastatic disease progression, immune cell regulation, and the recently-described neuroblastoma mesenchymal super-enhancer state (49). ARHGDIB and STAT1 are increased in models of breast cancer CTCs (50, 51) while STAB1 and TLR2 are increased in tumor-associated inflammatory cells in breast and colorectal cancer (52–55). Correspondingly increased expression of ARHGDIB (56, 57), OLFML2B (58–60), STAB1 (61) and TLR2 (62, 63) have been associated with disease relapse in most of the same cancers. Notably, STAB1 has been identified as a potential therapeutic target in neuroblastoma to block the tumorigenic effects of osteonectin (64).

Currently, prognostic and treatment decisions do not consider the gene expression profiles of metastatic cells, despite known genomic variations between CTCs, DTCs and primary tumors. There is also limited understanding of how this relates to CTC cell numbers, which we did not find to correlate with clinical prognostic variables. Indeed, MRD has also been identified in the bone marrow of children with low stage disease (64), supporting our proposed view that CTC gene expression may be more critical to overall disease phenotype than absolute cell numbers. Furthermore, significant gene expression changes have also been observed in CTCs and DTCs collected using density gradient methods corresponding to various states of clinical treatment failure and relapse (3, 48, 65), though these isolation approaches have not proven to be very efficient. Thus, the dysregulated genes identified in our study adds to the understanding of the altered gene expression landscape of neuroblastoma CTCs and DTCs.

Further clinical evaluation is required to better define the role of CTCs as markers of MRD and disease clearance, especially as CTCs were detected in relapsed patients whose routine disease markers were negative. It will be critical to establish if MRD-positivity in these patients may indicate potential upstaging in future. Suboptimal CTC enrichment may reflect limitations with current early-generation microfluidic technology, particularly for low volume blood samples and BMAs. In future, advanced single-cell capture and rare cell enrichment methods may facilitate the study of CTC biology *in vitro* and in CTC-derived xenograft models (6, 25, 66–68).

In summary, intact CTCs from peripheral blood of neuroblastoma patients enriched using label-free size-based cell separation expressed characteristic diagnostic markers. Putative gene signatures denoting CTCs associated with bone marrow metastases and latent disease relapse were identified and may facilitate further study of CTCs as a clinically-relevant and biologically-novel aspect of neuroblastoma MRD.

## Data availability statement

The original contributions presented in the study are included in the article/**Supplementary Material**, further inquiries can be directed to the corresponding author/s.

## Ethics statement

This study was reviewed and approved by SingHealth Duke NUS Centralised Institutional Review Board. Written informed consent to participate in this study was provided by the participants’ legal guardian/next of kin.

## Author contributions

Conceptualization: AL, ZC. Methodology: CA, SYS, TY, SL, YC, SMFA, SAS. Formal analysis: AL, MW. Investigation: CA, SYS, TY, SL, MW, YC, SMFA, SAS. Resources: AL, KC, TL, CL, ZC. Data curation: AL, CA, ST. Writing – original draft: AL, CA, ZC. Writing – review and editing: AL, WL, ZC. Visualization: AL, CA, MW. Supervision: ZC. Project administration: ST. funding Acquisition: AL, CL, ZC. All authors contributed to the article and approved the submitted version.

## Funding

This work was supported by SingHealth Duke-NUS Nurturing Clinician Scientists Scheme (04/FY2015/P2/11-A53), VIVA Foundation for Children with Cancer (VIVA-KKH Paediatric Brain and Solid Tumour Programme), and Children’s Cancer Foundation (Singapore Childhood Cancer Registry).

## Acknowledgments

The authors thank Candy Choo, Esther Hee, Zhang’e Choo, Khin Mar Cho, Josh Jie Hua Loh, Lorraine Yun Lin Yeo, Joyce Ching Mei Lam, Soh Hong Ang and Wen Di Lee.

## Conflict of interest

CL is a co-founder of Biolidics, Singapore.

The remaining authors declare that the research was conducted in the absence of any commercial or financial relationships that could be construed as a potential conflict of interest.

## Publisher’s note

All claims expressed in this article are solely those of the authors and do not necessarily represent those of their affiliated organizations, or those of the publisher, the editors and the reviewers. Any product that may be evaluated in this article, or claim that may be made by its manufacturer, is not guaranteed or endorsed by the publisher.

## Supplementary material

Supplementary Figure 1
**(A)** Representative image of enriched live NLF neuroblastoma cells (arrows) from ClearCell^®^ FX output, isolated using microfluidic single-cell capture device (scale bar: 20µm). **(B)** Expression of neuroblastoma gene markers and housekeeping genes in 15 captured single NLF cells, demonstrating highest expression of PHOX2B and TH, followed by GD2 synthase. **(C)** Relative gene expression of PHOX2B, TH and GD2 synthase in 7 neuroblastoma cell lines in comparison with non-neuroblastoma cell line AGS. NB: neuroblastoma; GC: gastric cancer.

Supplementary Figure 2 Heatmaps of Pearson z-scores of genes from the **(A)** PanCancer Pathway and **(B)** PanCancer Progression panels, of 17 patients at initial diagnosis, as well as normal controls of peripheral blood samples from 3 healthy subjects, showing segregation of cases with gene expression patterns similar or dissimilar to normal controls (red and green, respectively) on unsupervised hierarchical clustering. **(C)** Counts of atypical cells identified in each ¼ CTC-enriched fraction demonstrating immuno-positivity or negativity for PHOX2B and TH.

Supplementary Figure 3 Heatmap of log_2_ normalized counts of all 1490 cancer-related genes, clustered according to clinical and pathological variables and sites of metastases (Euclidean unsupervised hierarchical clustering). INPC: International Neuroblastoma Pathology Classification system. Clusters indicating upregulated genes in patients with bone marrow metastases and relapse are indicated in dashed lines.

Supplementary Figure 4
**(A)** MA (Bland–Altman) plots comparing means of normalized counts against the log fold change of genes from the PanCancer Pathway and Progression panels of enriched CTC fractions of 17 neuroblastoma patients. Colored points indicate genes with significant differential expression between patients with and without bone marrow metastases, at adjusted p-value threshold of 0.05. **(B)** Corresponding principal component analysis score plots of patients with and without bone marrow metastases.

Supplementary Table 1 Clinical and pathological characteristics of study patients.

Supplementary Table 2 Correlation of clinical variables with numbers of cells with cytologic atypia in peripheral blood CTC-enriched fractions at initial diagnosis (bold, P<0.05).
