# Supplementary figures and images for: Pro-metastatic and mesenchymal gene expression signatures characterize circulating tumor cells of neuroblastoma patients with bone marrow metastases and relapse
Source: Front Oncol. 2022 Sep 13;12:939460. doi: 10.3389/fonc.2022.939460 (PMC9513238; doi:10.3389/fonc.2022.939460)

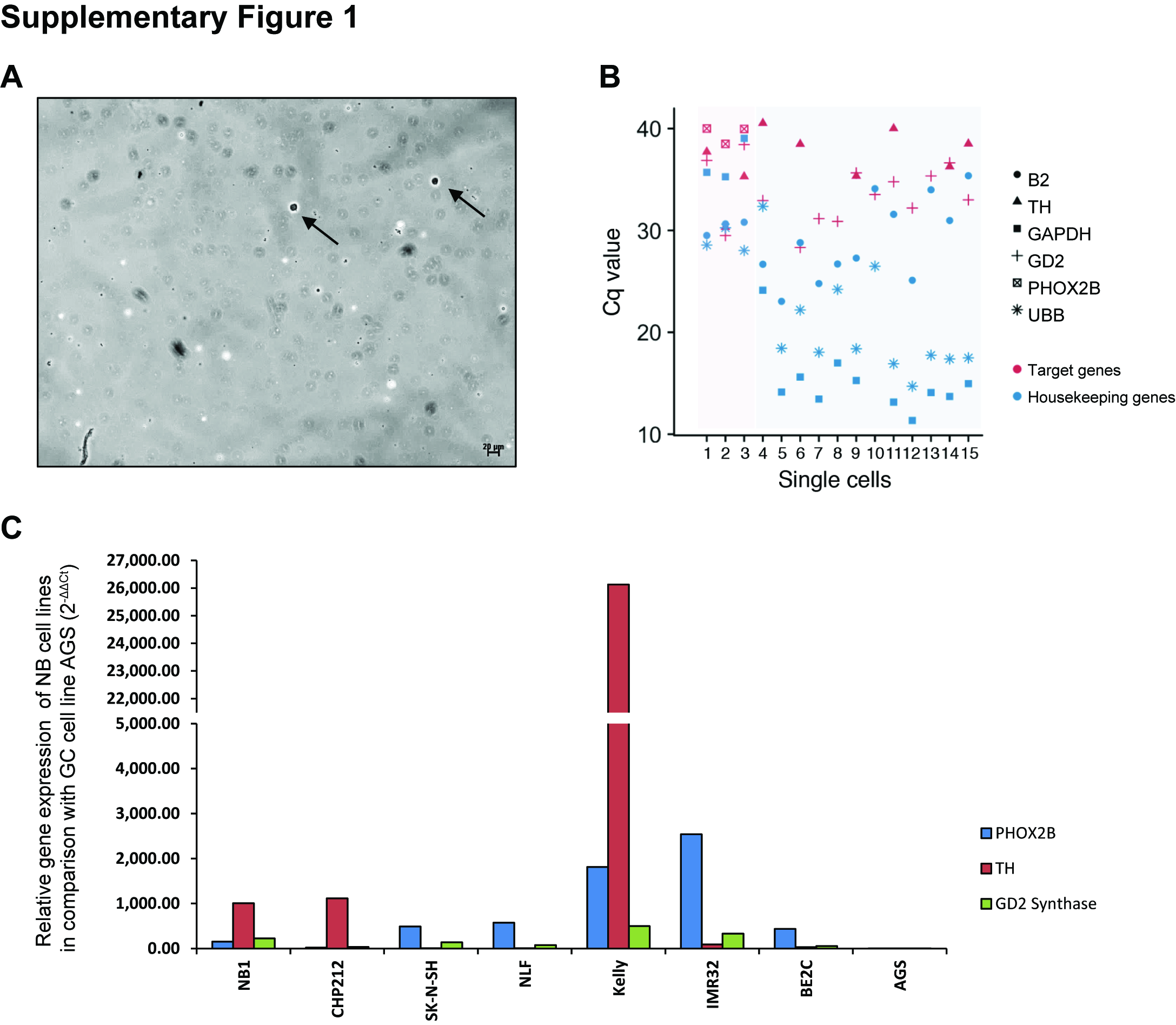

Supplement: Supplementary Figure 1 — (A) Representative image of enriched live NLF neuroblastoma cells (arrows) from ClearCell® FX output, isolated using microfluidic single-cell capture device (scale bar: 20µm). (B) Expression of neuroblastoma gene markers and housekeeping genes in 15 captured single NLF cells, demonstrating highest expression of PHOX2B and TH, followed by GD2 synthase. (C) Relative gene expression of PHOX2B, TH and GD2 synthase in 7 neuroblastoma cell lines in comparison with non-neuroblastoma cell line AGS. NB: neuroblastoma; GC: gastric cancer. [file Image_1.tif]

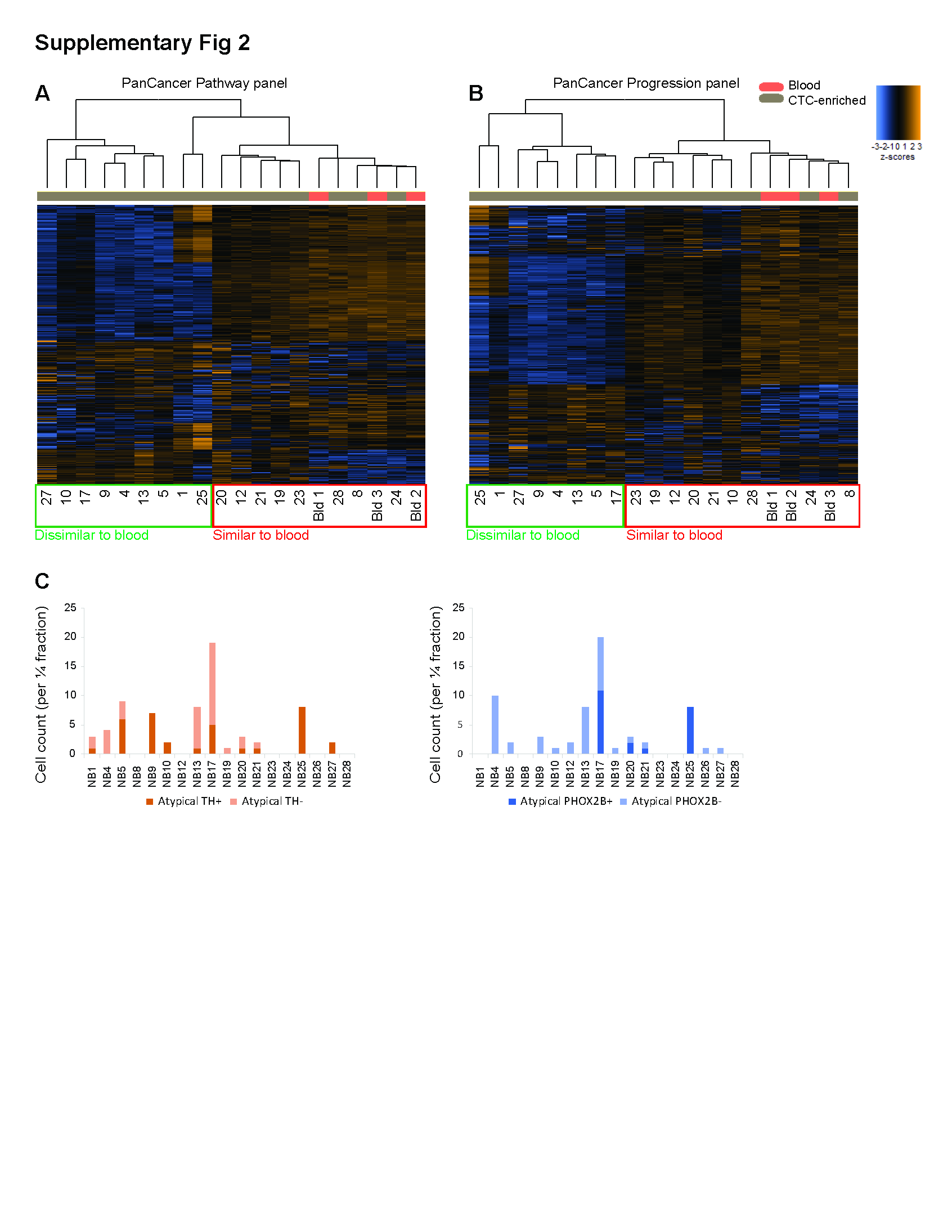

Supplement: Supplementary Figure 2 — Heatmaps of Pearson z-scores of genes from the (A) PanCancer Pathway and (B) PanCancer Progression panels, of 17 patients at initial diagnosis, as well as normal controls of peripheral blood samples from 3 healthy subjects, showing segregation of cases with gene expression patterns similar or dissimilar to normal controls (red and green, respectively) on unsupervised hierarchical clustering. (C) Counts of atypical cells identified in each ¼ CTC-enriched fraction demonstrating immuno-positivity or negativity for PHOX2B and TH. [file Image_2.tif]

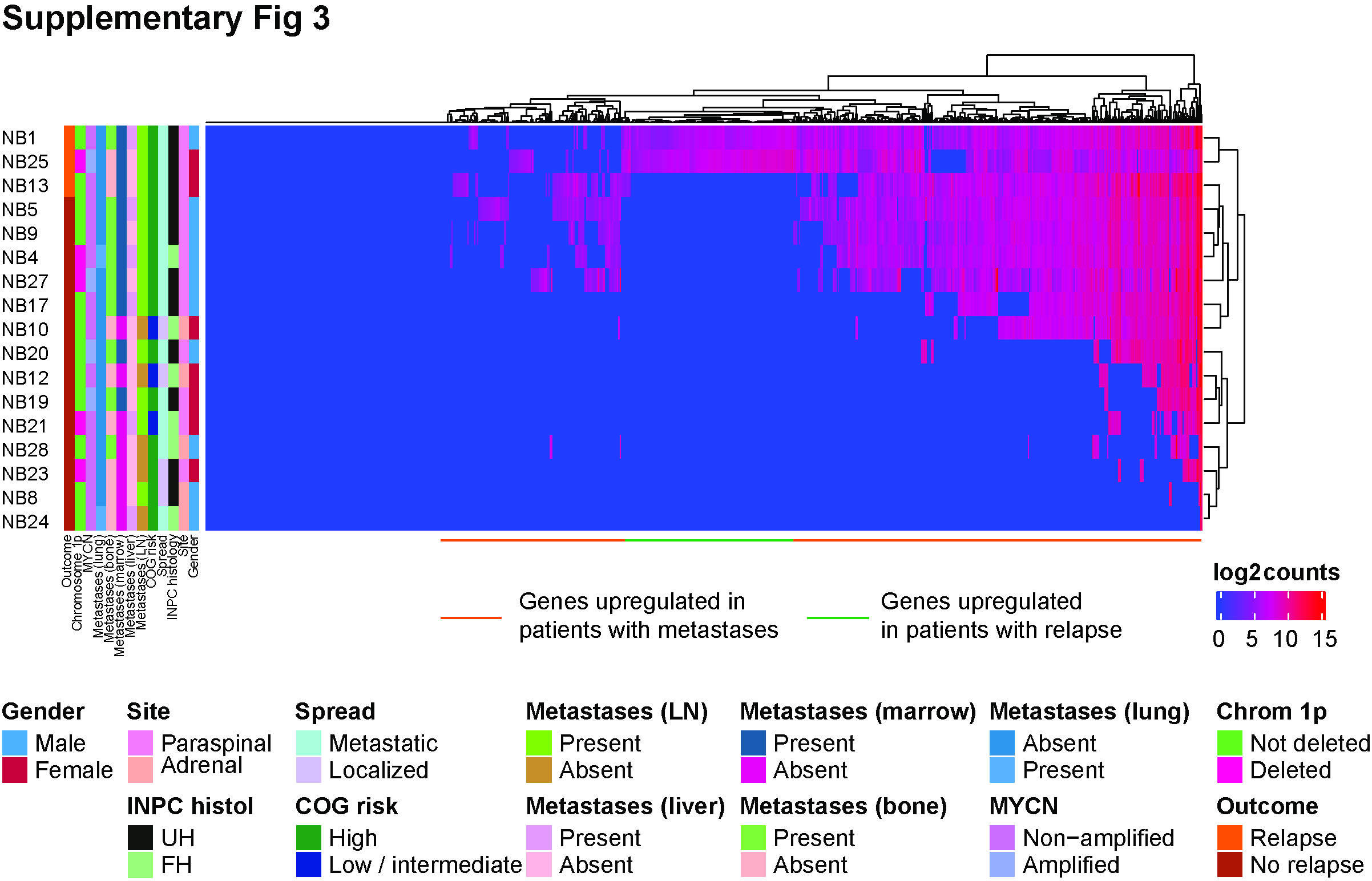

Supplement: Supplementary Figure 3 — Heatmap of log2 normalized counts of all 1490 cancer-related genes, clustered according to clinical and pathological variables and sites of metastases (Euclidean unsupervised hierarchical clustering). INPC: International Neuroblastoma Pathology Classification system. Clusters indicating upregulated genes in patients with bone marrow metastases and relapse are indicated in dashed lines. [file Image_3.tif]

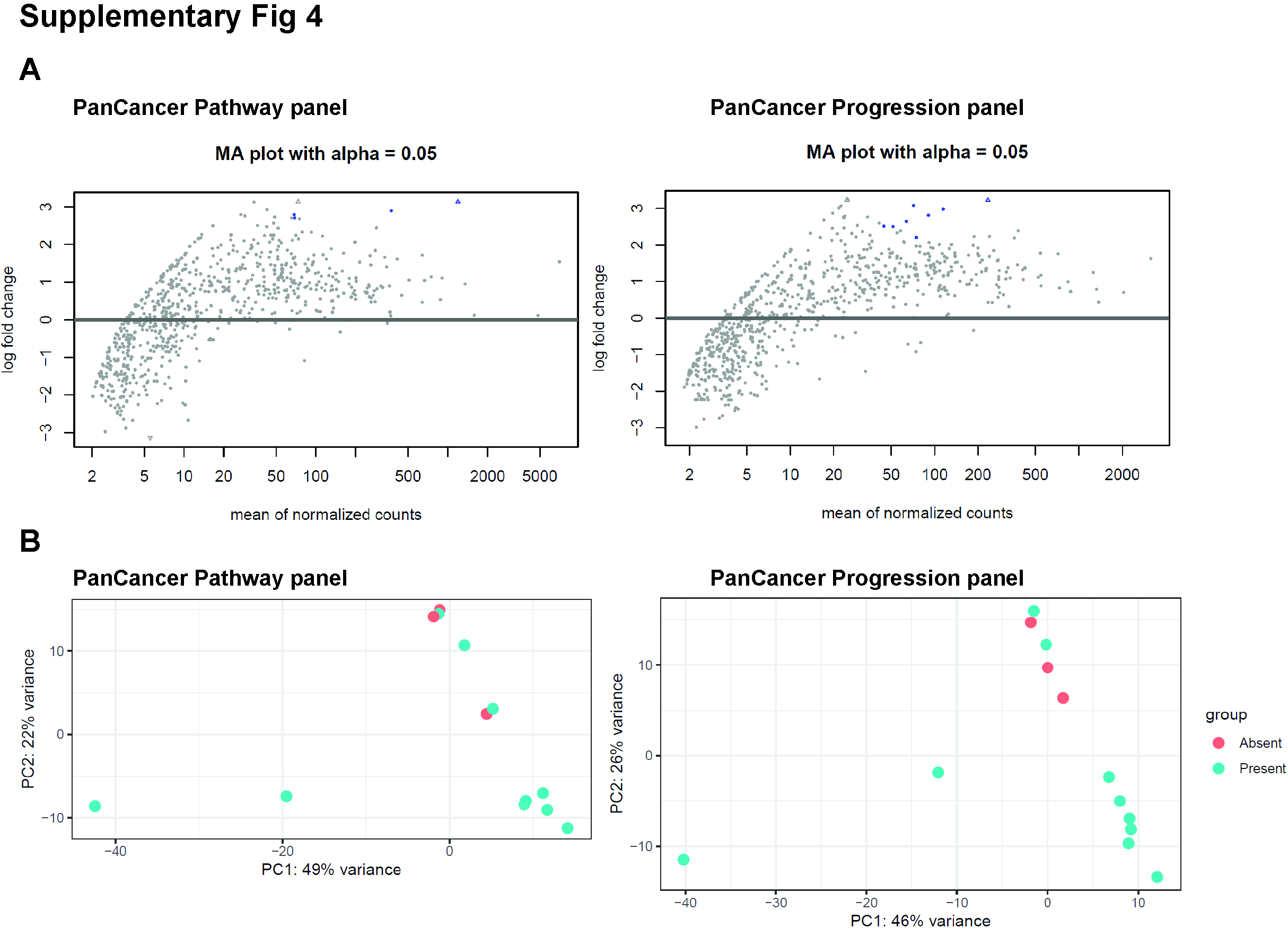

Supplement: Supplementary Figure 4 — (A) MA (Bland–Altman) plots comparing means of normalized counts against the log fold change of genes from the PanCancer Pathway and Progression panels of enriched CTC fractions of 17 neuroblastoma patients. Colored points indicate genes with significant differential expression between patients with and without bone marrow metastases, at adjusted p-value threshold of 0.05. (B) Corresponding principal component analysis score plots of patients with and without bone marrow metastases. [file Image_4.tif]
