# Supplementary material for: Pro-metastatic and mesenchymal gene expression signatures characterize circulating tumor cells of neuroblastoma patients with bone marrow metastases and relapse
Source: Front Oncol. 2022 Sep 13;12:939460. doi: 10.3389/fonc.2022.939460 (PMC9513238; doi:10.3389/fonc.2022.939460)
Supplement: Supplementary Table 1 — Clinical and pathological characteristics of study patients. [file Table_1.docx]

**Supplementary Table 1.** Clinical and pathological characteristics of study patients.

| **Study ID** | **Gender** | **Ethnic group** | **Age at diagnosis** | **Primary site** | **Histology** | **INSS** | **Risk** | **Metastatic sites** | | | | | **MYCN** | **1p** |
| --- | --- | --- | --- | --- | --- | --- | --- | --- | --- | --- | --- | --- | --- | --- |
|  |  |  |  |  |  |  |  | LN | Liver | Marrow | Bone | Lung |  |  |
| 1 | Male | Oriental | 5.03 | Adrenal | Undifferentiated neuroblastoma (UH) | Metastatic | High | Y | Y | Y | Y | N | Non-amp. | Not deleted |
| 4 | Male | Malay | 1.40 | Adrenal | Poorly differentiated neuroblastoma (FH) | Metastatic | High | Y | Y | Y | Y | Y | Non-amp. | Deleted |
| 5 | Male | Malay | 2.69 | Adrenal | Poorly differentiated neuroblastoma (UH) | Metastatic | High | Y | Y | Y | Y | N | Non-amp. | Not deleted |
| 8 | Male | Caucasian | 3.84 | Paraspinal | Metastatic neuroblastoma (UH) | Localized | High | Y | N | N | N | N | Non-amp. | Not deleted |
| 9 | Male | Oriental | 2.08 | Adrenal | Poorly differentiated neuroblastoma (UH) | Metastatic | High | Y | N | Y | Y | N | Non-amp. | Not deleted |
| 10 | Female | Oriental | 15.33 | Paraspinal | Ganglioneuroma (FH)* | Localized | Intermed. | N | N | N | N | N | Non-amp. | Not deleted |
| 12 | Female | Oriental | 4.59 | Paraspinal | Ganglioneuroma (FH)* | Localized | Low | N | N | N | N | N | Non-amp. | Not deleted |
| 13 | Female | Oriental | 17.79 | Adrenal | Differentiating neuroblastoma (UH) | Metastatic | High | Y | N | Y | N | N | Non-amp. | Not deleted |
| 17 | Male | Oriental | 31.23 | Adrenal | Metaastatic neuroblastoma (UH) | Metastatic | High | Y | Y | Y | Y | N | Non-amp. | Not deleted |
| 19 | Female | Oriental | 3.43 | Adrenal | Poorly differentiated neuroblastoma (UH) | Metastatic | High | Y | N | Y | Y | N | Amplified | Not deleted |
| 20 | Male | Oriental | 2.74 | Adrenal | Poorly differentiated neuroblastoma (UH) | Metastatic | High | Y | N | Y | Y | N | Amplified | Not deleted |
| 21 | Female | Malay | 0.39 | Adrenal | Poorly differentiated neuroblastoma (FH) | Metastatic | Intermed. | Y | Y | N | N | N | Non-amp. | Deleted |
| 23 | Female | Oriental | 2.12 | Adrenal | Poorly differentiated neuroblastoma (UH) | Localized | High | Y | N | N | N | N | Non-amp. | Deleted |
| 24 | Male | Oriental | 0.01 | Paraspinal | Poorly differentiated neuroblastoma (FH) | Metastatic | High | N | Y | N | N | Y | Non-amp. | Not deleted |
| 25 | Female | Malay | 0.91 | Adrenal | Undifferentiated neuroblastoma (UH) | Metastatic | High | Y | N | Y | N | N | Amplified | Deleted |
| 27 | Male | Malay | 4.65 | Adrenal | Poorly differentiated neuroblastoma (UH) | Metastatic | High | Y | N | Y | Y | N | Amplified | Deleted |
| 28 | Female | Oriental | 6.09 | Paraspinal | Differentiating neuroblastoma (FH) | Metastatic | High | Y | N | N | Y | N | Non-amp. | Not deleted |

INSS: International neuroblastoma staging system

LN: lymph nodes

FH: Favorable histology

UH: Unfavorable histology
