# Supplementary material for: Pro-metastatic and mesenchymal gene expression signatures characterize circulating tumor cells of neuroblastoma patients with bone marrow metastases and relapse
Source: Front Oncol. 2022 Sep 13;12:939460. doi: 10.3389/fonc.2022.939460 (PMC9513238; doi:10.3389/fonc.2022.939460)
Supplement: Supplementary Table 2 — Correlation of clinical variables with numbers of cells with cytologic atypia in peripheral blood CTC-enriched fractions at initial diagnosis (bold, P<0.05). [file Table_2.docx]

**Supplementary table 2.** Correlation of clinical variables with numbers of cells with cytologic atypia in peripheral blood CTC-enriched fractions at initial diagnosis.

| **Variable** | | **Patients (n)** | **Total cells with cytologic atypia (n)** | | |
| --- | --- | --- | --- | --- | --- |
|  |  |  | **Mean (S.D.)** | **t** | ***P*** |
| Primary tumor site | Adrenal | 12 | 10.33 (10.63) | 1.67 | 0.12 |
|  | Paraspinal | 5 | 1.25 (1.5) |  |  |
| INPC histology | Unfavorable | 11 | 9.64 (11.36) | 0.93 | 0.37 |
|  | Favorable | 6 | 4.6 (5.46) |  |  |
| Spread | Non-metastatic | 4 | 1.25 (1.5) | -1.67 | 0.12 |
|  | Metastatic | 13 | 10.33 (10.63) |  |  |
| Metastases (lymph nodes) | Yes | 14 | 9.54 (10.57) | 1.25 | 0.23 |
|  | No | 3 | 1.67 (1.53) |  |  |
| Metastases (liver) | Yes | 6 | 11.83 (14.3) | 1.19 | 0.26 |
|  | No | 11 | 5.8 (6.12) |  |  |
| Metastases (bone marrow) | Yes | 10 | 12 (10.89) | 2.31 | **0.04** |
|  | No | 7 | 1.5 (1.76) |  |  |
| Metastases (bone) | Yes | 9 | 11 (12.12) | 1.19 | 0.25 |
|  | No | 8 | 5.13 (6.88) |  |  |
| Metastases (lung) | Yes | 2 | 7 (9.9) | -0.16 | 0.88 |
|  | No | 15 | 8.21 (10.36) |  |  |
| COG risk | Low / int. | 3 | 3 (1) | -0.97 | 0.35 |
|  | High | 14 | 9.23 (10.8) |  |  |
| MYCN | Amplified | 4 | 6.75 (6.4) | -0.29 | 0.77 |
|  | Non amplified | 13 | 8.5 (11.14) |  |  |
| 1p | Deletion | 5 | 7.4 (7.13) | -0.17 | 0.87 |
|  | No deletion | 12 | 8.36 (11.36) |  |  |
| Relapse | Relapse | 3 | 11.67 (7.51) | 0.68 | 0.51 |
|  | No relapse | 14 | 7.23 (10.55) |  |  |

INPC: International Neuroblastoma Pathology Classification; COG: Children’s Oncology Group.
